# Understanding the complexities of financial support for students in grant-funded STEMM training programs

**DOI:** 10.3389/feduc.2025.1445151

**Published:** 2025-04-29

**Authors:** Ana L. Romero, Krystle P. Cobian, Patricia A. Martín

**Affiliations:** 1 School of Education and Information Studies, University of California, Los Angeles, Los Angeles, CA, United States,; 2 David Geffen School of Medicine, University of California, Los Angeles, Los Angeles, CA, United States

**Keywords:** STEMM education, financial support, financial aid, Social Cognitive Career Theory, research training, STEMM intervention program, scholar program

## Abstract

**Introduction:**

There are lessons to be learned from campuses providing financial support to students involved in grant-funded science, technology, engineering, mathematics, and medicine (STEMM) training initiatives. We examine students’ perspectives on financial support from the Building Infrastructure Leading to Diversity (BUILD) program, funded by the National Institutes of Health.

**Methods:**

We analyzed qualitative data collected from 122 BUILD undergraduate participants during site visits to the 10 NIH-sponsored BUILD programs using generic qualitative inquiry.

**Results:**

Using Social Cognitive Career Theory (SCCT) to guide qualitative data analysis, we found that students perceived BUILD funding to reduce financial stress and increase access to career training; however, the impact of aid was hampered by limitations in funding and financial aid processes.

**Discussion:**

Findings from this study reveal that financial support from BUILD often facilitated college entry and participation in biomedical research training experiences for students. While students viewed financial support as beneficial to their academic and professional trajectory, they also noted challenges with financial aid processes on campus. This study has implications for federal funding agencies, foundations, and higher education institutions, specifically in developing innovative disbursement processes to improve support and to reduce unintended harmful consequences for student recipients.

## 1 Introduction

Degree attainment levels in science, technology, engineering, mathematics, and medicine^1^ (STEMM) continue to fall short, especially among underrepresented student populations. In 2012, a report from the President’s Council of Advisors on Science and Technology [PCAST] (2012) recommended a concerted effort to reduce STEMM attrition in colleges so as to increase the number of STEMM professionals and meet the national need for researchers in scientific areas of expertise. Aimed at understanding college student attrition in STEMM disciplines, a statistical report commissioned by the U.S. Department of Education examined a cohort of students who began postsecondary education in 2003–2004 through 2009 and found that the percentage of Pell Grant recipients who dropped out of college was higher than that of non-Pell Grant recipients (25 vs. 18 percent) (Chen, 2013). Since then, national efforts have focused on better understanding the experiences of students with respect to race and ethnicity, socioeconomic status, gender, disability, and first-generation status to address equity gaps in their participation and success in college, and ultimately in the STEMM workforce (Committee on Equal Opportunities in Science and Education, 2022; National Academies of Sciences, Engineering, and Medicine[NASEM], 2023). Unfortunately, disparities still persist in STEMM participation. For example, an examination of national data reveal that Latine^2^, Black, and American Indian or Alaska Native individuals collectively accounted for 37% of 18–34-year-old individuals in 2021, yet they only make up 26% of science and engineering bachelor’s degree earners in 2020 (National Center for Science and Engineering Statistics [NCSES], 2023).

Agencies such as the National Institutes of Health (NIH), National Science Foundation (NSF), Howard Hughes Medical Institute (HHMI), and Alfred P. Sloan Foundation have provided multiple funding opportunities to support undergraduate education and promote opportunities for individuals from groups who are underrepresented in the STEMM workforce, for example low-income and underrepresented racial/ethnic groups (Estrada et al., 2016). These and other STEMM intervention programs vary in their purpose and approach but have a common thread of supporting underrepresented students pursuing STEMM degrees (Lane et al., 2020). These programs commonly use evidence-based activities such as pre-college support, academic bridge programs, living-learning communities, research participation, supplemental education, and mentorship to support the retention of underrepresented students in STEMM (Rincon and George-Jackson, 2016; Tsui, 2007). Often, such programmatic efforts also include pairing financial support (e.g., stipends, tuition discounts, undergraduate research hourly pay) with academic interventions with an aim to reduce financial barriers to STEMM degree completion.

Financial support has been shown to have a positive impact on various student outcomes. Need-based aid contributes to college selection (Foltz et al., 2014) and to faster completion rates of bachelor’s degrees (Anderson et al., 2023). Studies focused on the impact of merit-based aid STEMM programs (e.g., S-STEM, Meyerhoff Program, Equitable Pathways to STEM Graduate Education) have found that many factors in addition to financial support, such as other academic support, mentoring, community building, and psychosocial program elements, contribute to student retention in STEMM (Foltz et al., 2014; Maton et al., 2009; Wilson et al., 2012; Wohlgemuth et al., 2007) especially for students from underrepresented backgrounds and with financial need (Chang et al., 2016; Wolniak and Pascarella, 2007; Wright et al., 2021). While most merit-based or need-based aid studies quantitatively examine long-term outcomes such as time-to-degree or baccalaureate completion rates, few studies qualitatively examine how financial support may impact STEMM students along their career pathways. While the literature on the impact of financial aid suggests its importance in contributing to both college access and completion (Bettinger, 2004; Goldrick-Rab et al., 2016), specific outcomes related to use of financial aid by students in STEMM fields warrants further investigation. Additional research is necessary to understand the full impact of the financial aid investments made by funding agencies and STEMM intervention programs. A qualitative approach can offer a more nuanced understanding of benefits and challenges of financial support that may be obscured by quantitative studies.

This study examines undergraduate students’ experiences regarding financial support received in conjunction with their participation in the Building Infrastructure Leading to Diversity (BUILD) initiative, a STEMM intervention program funded by the National Institutes of Health (NIH) and evaluated through the Enhance Diversity Study (EDS). Specifically, this study is interested in the role of financial support from a STEMM training program on the career pathway of undergraduate students and their persistence. We address the following questions: How do BUILD-exposed students describe the importance of funding in navigating their college experience? Through examination of financial support as a contextual influence, how do undergraduate student participants in the BUILD program perceive the role of financial support in shaping their interests, goals, and actions? Insights from this study are significant for funders, researchers, and program administrators interested in and/or responsible for implementation of STEMM training programs.

## 2 Literature review

In this section, we provide an overview of research on the role of financial support in student outcomes and its influence on STEMM. We also provide detailed information on the BUILD research training program.

### 2.1 Role of financial support in higher education

The role of financial support in student outcomes in higher education has been studied extensively. Aid that does not require repayment, such as merit-based aid (e.g., scholarships, grants, or tuition discounts) and need-based aid (e.g., Pell Grants, scholarships, grants determined by financial eligibility), is of particular interest because individuals who participate in STEMM intervention programs often receive a mix of both types of financial support. Need-based financial aid has been shown to increase college access by increasing college enrollment and attendance rates (Dynarski and Scott-Clayton, 2013). Studies examining college persistence and completion for recipients of need-based grant aid have largely found positive results. For example, Alon (2011) found need-based aid had a positive impact on college persistence among low- and middle-income students. Other studies estimating the causal effect of need-based aid, such as Pell Grants, suggest that this type of financial aid may reduce dropout rates (Bettinger, 2004). Similarly, Eng and Matsudaira (2021) found that an increase in the amount of Pell Grant aid awarded to a student has a small but positive effect on the probability of completing a college degree within 6 years of enrolling for dependent students and independent students with dependents. Focusing on the policy implications of need-based aid, Sneyers and De Witte (2018) conducted a meta-analysis of 10 quasi-experimental and experimental studies and found a 2.5% increase in need-based aid recipients’ enrollment, retention, and graduation. Much of the literature on need-based aid suggests that more than this type of financial support is required to ensure successful college outcomes and that more research is needed to explore long-term effects of need-based aid (Bettinger, 2004).

Researchers have also noted the impact of merit-based aid on college persistence and completion (Dynarski, 2004; Dynarski and Scott-Clayton, 2013; Patel and Richburg-Hayes, 2012; Scott-Clayton, 2011). Previous literature has discussed the widespread impact of merit-based aid programs, many of which are state-led and sponsored, such as Georgia’s HOPE program and California’s Cal Grant program, on college attendance (Dynarski, 2004), college-going rates of Black and African American students (Henry and Rubenstein, 2002), and degree attainment (Bettinger et al., 2016). Using data from a small, public liberal arts college in New Jersey, Olbrecht et al. (2016) found that increasing institutional merit-based financial aid had positive effects on student retention. They suggest even small amounts of merit aid can have large gains for student retention (Olbrecht et al., 2016). Financial aid scholarships and grants with specific academic eligibility requirements have been found to increase persistence and completion (Dynarski and Scott-Clayton, 2013; Patel and Richburg-Hayes, 2012; Scott-Clayton, 2011). Specifically, academic and performance requirements of merit-based aid contribute to degree completion by incentivizing students to excel academically and reducing their time-to-degree (Scott-Clayton, 2011). These studies foreground the importance of need-based and merit-based aid on college retention and success. However, a study examining the effects of loss of merit aid that employed a regression discontinuity design using data from the Tennessee HOPE scholarship found heterogeneous results by subgroups (Cummings et al., 2022). These findings suggest loss of eligibility at the first renewal checkpoint (e.g., receiving below a cumulative 2.75 GPA) leads to a higher probability of “stopping out” (i.e., leaving their program temporarily or permanently dropping out) among higher-income White students and an increased transfer to a community college for low-income Black students (Cummings et al., 2022). Results from this study illustrate the need for additional research to better understand and discern the role of financial aid on students’ academic trajectory.

### 2.2 Role of financial support in STEMM participation

Financial support is associated with entry into college, especially among underrepresented and low-income students interested in STEMM. Foltz et al. (2014) found that financial support can often influence institutional choice for entering underrepresented STEMM students as they weigh financial aid packages and college-related costs in their college decisions. For example, a study on community college women in STEMM majors found students chose to attend a community college due to the lower cost of attendance (Packard et al., 2011). Students in STEMM from low-income backgrounds face multiple challenges of paying for college while maintaining good grades to succeed in demanding majors (Anderson et al., 2023; Sjoquist and Winters, 2015; Zhang, 2011). A study examining the causal impact of need-based grant aid from the Wisconsin Scholars Grant on students’ major choice found an increase in the share of students declaring a major in a STEM field (Anderson et al., 2023). Financial challenges can, therefore, influence where students go to college and potentially impact their ability to pursue a STEMM degree (Holland Zahner, 2023).

Financial support is also influential in increasing access to STEMM-related activities for underrepresented and low-income students. Economic disparity can “impact URM [underrepresented racial minorities] STEM[M] students’ career trajectories” (Estrada et al., 2016, p. 7), because URM students, especially those from low socioeconomic status, are more likely to work during college and thus have less time for studying, obtaining research experience, participating in STEMM organizations, and attending summer STEMM preparation programs (Estrada et al., 2016). Estrada et al. (2016) suggest that financial support can mitigate economic disparities for underrepresented minority groups by increasing their access to such training opportunities. For example, the financial support provided by the Meyerhoff Scholars Program, a merit-based program open to high-achieving high school seniors of all backgrounds committed to increasing the representation of minorities in science and engineering, was a major factor in students’ decision to apply and participate in the program (Stolle-McAllister et al., 2011). For context, the program provides scholarships that range from $5,000 to 22,000 per year to undergraduate students as well as structured professional development activities to prepare Scholars for graduate study and a career in a STEMM field. Additionally, financial support from the program enabled students to participate in internships and conferences (Stolle-McAllister et al., 2011). Funding from STEMM intervention programs, such as the Meyerhoff Program, enables students to participate in activities that contribute to their professional development and career preparation (Cobian et al., 2024; Stolle-McAllister et al., 2011). It is important to note that, along with funding, comes significant academic and social support in these types of scholar programs.

### 2.3 Role of financial support on STEMM persistence

Underrepresented student populations and low-income students typically persist in STEMM at lower rates than their well-represented, higher-income peers (Castleman et al., 2018; Cromley et al., 2016; Estrada et al., 2016; Fenske et al., 2000; Gibson et al., 2020). Several factors contribute to these persistent inequities. For example, STEMM majors tend to average a longer time-to-degree than degree completers in other fields or switch out of STEMM majors altogether (Castleman and Long, 2016; Chen, 2013), which can add additional strain on students who already struggle with college affordability. Additionally, students of color experience several forms of marginalization significantly more often than nonminority students, including lack of access to resources that position them for college success. In practice, marginalization occurs via perceptions and resulting actions from others within STEMM (e.g., racism and exclusionary practices in the classroom and lab settings) (McGee, 2019), and many students who identify as first-generation college students and members of racial and ethnic group underrepresented in STEMM must overcome barriers created by having fewer social networks and academic socialization experiences (Allaire, 2019) and the stress of financial precarity.

Students persist in STEMM majors at significantly higher rates when they receive significant levels of financial support (Cromley et al., 2016; Estrada et al., 2016; Tsui, 2007; Wang, 2013; Whalen and Shelley, 2010; Wilson et al., 2012). Whalen and Shelley (2010) found that students who are underrepresented in STEMM fields were less likely to be retained in STEMM or graduate within 6 years compared to students who are traditionally well-represented in STEMM majors. However, when paired with mentoring and training, Wilson et al. (2012) found that financial support contributed to the retention of low-income undergraduate STEMM students. Similarly, Packard et al. (2011) found that financial barriers, including having to work while studying, deterred women who transferred from community colleges to 4-year institutions from completing their STEMM degree. Pairing financial support with program activities that support STEMM career development and increase students’ sense of belonging in the field contributes to diversifying students in STEMM (Tsui, 2007) and is critical for persistence at multiple levels of the educational pathway (McGee, 2019).

### 2.4 Relationship between college funding and long-term STEMM outcomes

There is a great deal of interest in research examining mid-and long-term outcomes of financial support for individuals with STEMM career aspirations. Studies have focused on examining retention and degree attainment rates and their influence on graduate school entry. Pairing financial support with other support, such as academic enrichment, can improve STEMM retention (Gibson et al., 2020), STEMM degree attainment (Castleman et al., 2018), and the pursuit of graduate education (Maton et al., 2009). Anderson et al.’s (2023) longitudinal assessment of the privately funded, need-based Wisconsin Scholars Grant (WSG) adds nuance to the literature. Their findings suggest that while the WSG did not increase the volume of bachelor’s degree attainment, WSG recipients earned their bachelor’s degrees faster and demonstrated a small participation-rate increase in STEMM fields compared to non-WSG peers (Anderson et al., 2023).

STEMM programs that offer financial and other sources of support have been shown to improve student outcomes. An evaluation of two NSF-funded STEMM intervention programs designed to support students from financially disadvantaged backgrounds at Louisiana State University (LSU) showed significant increases in 6-year graduation rates of students involved in the programs compared to LSU science majors who were not involved. Myers and Pavel (2011) used longitudinal data from both recipients and non-recipient of the Gates Millennium Scholarship (GMS) to analyze the effect of being a GMS scholar and STEMM major on entrance into STEMM graduate degree programs. They found that GMS scholar status increases the odds of enrolling in a STEMM graduate program regardless of students’ undergraduate majors, speaking to the benefits of programs that offer financial support and other resources on postgraduate STEMM outcomes. Given that college debt often deters students with a STEMM bachelor’s degree from seeking graduate education, programs that offer undergraduate financial support can encourage more underrepresented students to seek postgraduate opportunities in STEMM (Malcom and Dowd, 2012).

### 2.5 Challenges of financial support in STEMM intervention programs

Although studies point to many benefits of financial support, some have highlighted students’ continued concerns and challenges. Students with a strong college interest may lack understanding of the financial aid process (Foltz et al., 2014). Providing adequate information to prospective and current college students, especially underrepresented students, is vital to inform their decision-making at college entry and throughout their undergraduate years. Studies have shown that financial concerns are also associated with lower participation rates in undergraduate biomedical training programs (Eagan et al., 2024), which demonstrates the need to make funding available to students.

Objective and perceived bureaucratic challenges at the institutional level, including challenges with the financial aid process, can also influence student outcomes. For example, students who encounter difficulties navigating university-related issues, including help with receiving promised financial aid, have lower intentions of pursuing a higher degree (Slovacek et al., 2011). Indeed, pairing financial support with educational support “can be as effective or more effective than just delivering more aid through a complicated system” (Anderson, 2020, p. 10) – particularly for underrepresented students whose entry into higher education begins at community colleges and technical colleges (Dowd, 2007; Wang, 2009). More research is needed to understand the role of financial support as one component of postsecondary STEMM intervention programs.

### 2.6 Building infrastructure leading to diversity (BUILD) initiative

To understand how financial support shapes career trajectories, as part of the Enhance Diversity Study (EDS) evaluation, we examined efforts from a large-scale investment in STEMM research training known as the BUILD initiative. Funded by NIH from 2014 to 2024, BUILD awards provided funding for implementing and evaluating a range of programs and structural changes aimed at engaging and increasing the retention of individuals from diverse backgrounds in the biomedical research workforce (McCreath et al., 2017). In total, ten BUILD sites nationwide received funding for student training, faculty development, and institutional capacity-building activities (Hurtado et al., 2017).

The BUILD initiative’s student training programs varied according to each site’s proposed plans. Each BUILD site implemented their own recruitment and selection process; however, minimum criteria for BUILD Scholar applications was consistent across sites. Applicants were required to enroll full-time, be in a biomedical or STEMM-related major, have earned a minimum 2.75 college GPA or 3.0 high school GPA, demonstrate an interest in pursuing graduate studies in a biomedical or related discipline, and show an interest in biomedical research (Eagan et al., 2024). Most sites provided various forms of cohort-based activities, professional development, mentorship, and research experience to BUILD undergraduate scholars/research trainees. BUILD often provided these students with funding to support conference travel and research dissemination. Additionally, some students also received funds to cover at least a portion of tuition and fees. Each site determined how to distribute the financial support to BUILD students as well as funding amounts. All sites developed an undergraduate program designed to provide the highest “dosage” of program interventions to BUILD Scholars and partial funding and program support to BUILD Associates. For example, Scholars may have been offered 60% tuition coverage and a monthly stipend and professional development activities, whereas associates may have only been provided with professional development activities and conference travel funding. Our study aims to understand how BUILD financial support, within the context of the larger BUILD initiative, shaped students’ educational and biomedical career aspirations.

## 3 Theoretical framework

This study uses social cognitive theory (Bandura, 1977, 1986, 1997), which posits that individuals both influence and are influenced by their environment. Specifically, we draw from the latter stages of Social Cognitive Career Theory (SCCT), which focuses on the interactions of contextual influences with students’ career interests, goals, and actions (Lent et al., 2002). Derived from social cognitive theory, SCCT assumes that career development and career trajectories are shaped by the interactions between three domains: individual attributes (e.g., interests), learning and socialization experiences, and contextual influences (i.e., environmental factors) (Lent, 2020). Lent et al. (2002) argue that these early experiences interact with students’ interests, goals, and actions, ultimately shaping students’ career outcomes.

Of particular interest in our study is how contextual influences within the SCCT model shape students’ interests, goals, and actions. We conceptualize the BUILD program’s financial support as a primary contextual influence, similar to a study conducted by Wang (2013), while also keeping in mind that program scholars also receive other forms of support (e.g., learning communities, mentorship, research training experiences) through BUILD. According to SCCT, career development is influenced by both objective and perceived environmental factors (Lent et al., 2000). In other words, the effect of a particular objective factor, such as financial support, depends on an individual’s perception and response to it (Vondracek et al., 1986). Contextual influences that are proximal to choice behavior can inform career decisions at different critical junctures (Lent et al., 2002). Lent et al. (2002) posit that contextual experiences can influence the connection between interests and goals as well as the connection between goals and actions. According to the theory, people who face few barriers and are offered a high degree of support have an easier time navigating their goals and choosing their preferred course of action (Lent et al., 2002). There is typically an assumption, supported by evidence discussed earlier, that providing financial support via STEMM intervention programs will reduce barriers to obtaining key experiences and academic milestones to persist on a STEMM career pathway. How financial support is perceived by student recipients to shape STEMM career aspirations is not as well studied, however.

Most prior studies have taken a quantitative approach to applying SCCT to undergraduate STEMM career development (Byars-Winston and Rogers, 2019; Hazari et al., 2010; Inda et al., 2013; Lent et al., 2008). While the body of literature employing SCCT has examined barriers, there is a lack of qualitative approaches to applying a SCCT framework to career development (Lent et al., 2000; Wang et al., 2022). A qualitative approach to evaluating the experiences of students in STEMM intervention programs can discover nuances in the interaction of the contextual influence (i.e., environment) with career interests, goals, and actions. In our study, we examine financial support as the contextual factor that influences STEMM students’ experiences.

## 4 Methods

We used qualitative data collected during site visits to the 10 NIH-sponsored BUILD programs. These sites include California State University, Long Beach (CSULB); California State University, Northridge (CSUN); Morgan State University (MSU); Portland State University (PSU); San Francisco State University (SFSU); University of Alaska, Fairbanks (UAF); University of Detroit Mercy (UDM)/Wayne State University (WSU); University of Maryland, Baltimore County (UMBC); the University of Texas at El Paso (UTEP), and Xavier University of Louisiana (XULA).

This study used generic qualitative inquiry to examine how BUILD students made sense of the funding they received from the BUILD program and the perceived influence it had on their respective STEMM career pathways. Generic qualitative inquiry is appropriate when studies do not follow specific forms of qualitative methodology, such as narrative inquiry or phenomenology (Caelli et al., 2003; Kahlke, 2014). Because the data were originally collected to explore and understand each site’s program implementation and this study focuses specifically on students’ experiences, we analyzed BUILD students’ narratives to understand their perspectives on the relationship between funding and students’ career interests, goals, and actions.

### 4.1 Participants

Students were recruited from the pool of BUILD Scholars and Associates to participate in focus groups and discuss their experiences. In order to participate in BUILD, students, regardless of year of study (first-years, sophomores, juniors, or seniors), were selected from eligible majors: life sciences (e.g., biology), engineering (e.g., chemical, mechanical), health professions (e.g., nursing, public health), physical sciences (e.g., biochemistry, chemistry), and social sciences (e.g., psychology). Over half (56%) of students were biology and life science majors. The remainder of student participants reported majors in social sciences (16%), health professions (14%), engineering (7%), physical sciences (2%), computer science (2%), and 7% did not share their major^3^. Nearly a quarter (24.6%) of participants were classified as a first-year student or sophomore, 41% were juniors or seniors, 4.9% were recent alumni who participated in BUILD as undergraduate students, and 29.5% did not provide their class standing (more information about the grouping of students is described in the following section on data collection). BUILD participants typically identified with one or more underrepresented groups in STEMM (e.g., race/ethnicity, gender, income, disability).

Students selected into BUILD were often, but not always, offered financial support. The financial support varied across students within and across institutional contexts. For example, several students received a stipend as compensation for their work in undergraduate research experiences (e.g., working in research labs). Additionally, many students received additional funding in the form of tuition remission. BUILD also provided financial support for conference travel and research dissemination. The financial support was intended to relieve financial barriers and contribute to underrepresented students’ persistence in STEMM.

### 4.2 Data collection

A research team conducted 2-day site visits at each of the 10 BUILD programs. Data were collected in two phases, with the first five site visits occurring in the first half of 2017 and the other five site visits occurring in the fall of 2018 (Moses et al., 2020). The site visits took place in the early stages of the initiative but also with enough time for programs to become established. The rationale for this provided an opportunity for feedback to each site, and also to begin to see potential problems arising across the campuses. Data collection primarily consisted of semi-structured interviews and focus groups. Interview participants in the larger research project included program administrators, senior administrators, faculty, and students. Individual and focus group interviews were audio recorded, and they were transcribed manually via a transcription service and stored in a secure database. All data protection measures were in compliance with the Institutional Review Board (IRB). Interviews were then uploaded, coded, and analyzed using Dedoose which is a web-based qualitative and mixed-methods software. Additional data consisted of observation notes, analytic memos, and documents that explained how each BUILD site implemented program objectives (Moses et al., 2020). The research team wrote observation notes during site visits that described physical spaces impacted by BUILD and information that helped contextualize the site. Analytic memos were written by team members who engaged in data coding and analysis. Documents consisted of reports (e.g., debrief reports) and were stored in a secure database. Observation notes, analytic memos, and documents were not analyzed for this study, but they were used to understand site context and confirm findings. The site visits were exploratory in nature and primarily focused on understanding the BUILD initiatives, program implementation, and participant experiences.

For this study, we limited the scope of the analysis to data collected from student focus groups during the site visits. The study team facilitated 21 semi-structured student focus group interviews with 122 participants across the 10 BUILD sites. Two focus groups were held at each institution and students were invited to participate in one of the focus groups except at UAF where all BUILD students were invited to one joint focus group. On each campus, one focus group was intended for early BUILD Scholars or Associates (first-year students and sophomores) and the other with upperclassmen (juniors and seniors). This design intended to divide focus groups into students who were newer to the institution versus BUILD Scholars who had already completed introductory courses. While this design helped differentiate experiences between each class grouping, students were often affected similarly by campus and programmatic financial support practices. The duration of these focus groups averaged between 60 and 90 minutes. Participants were asked questions regarding their motivation for participating in BUILD, the impact of BUILD activities, and the challenges they experienced. We used transcripts from the student focus group interviews, demographic questionnaires, and researcher notes.

### 4.3 Data coding and analyses

After site visits were completed, the research team took an inductive and deductive coding approach to analyze interviews (Moses et al., 2020). The team developed a codebook that accounted for the four levels of data collected at each BUILD site: institutional, faculty, student, and program. Codes were derived deductively by applying concepts from the literature as well as outcomes of interest identified by the NIH Diversity Program Consortium (DPC) (Moses et al., 2020). Inductive coding included open coding, which led to additional codes that the research team derived after reviewing the data. The research team used Dedoose to calculate interrater reliability using a Pooled Cohen’s Kappa coefficient (Cohen, 1960). Landis and Koch (1977) indicate a kappa score from 0.81 to 1.00 is considered “almost perfect” (p. 165); therefore, the research team determined a score above 0.80 would be acceptable. The research team achieved a score above 0.90 (Moses et al., 2020) and determined the agreement level was sufficient to begin independent coding.

For this study, the research team identified student-level coded text segments from the larger codebook that were relevant to understanding the complexities of student finances. We focused on these previously coded segments and conducted additional iterations of open and axial coding to engage with the qualitative data while also considering the study’s theoretical framework emphasizing student financial support as a contextual experience. Last, the team utilized documents collected during site visits and information publicly available on each BUILD website to supplement the team’s familiarity with each site’s program and key features, and to triangulate findings.

### 4.4 Trustworthiness

We took several steps during data collection and analysis to ensure trustworthiness. The research team held debrief meetings with BUILD program leaders at the end of each site visit to share initial findings and observations. Each site was provided a debrief report that included the research team’s initial findings. Program leaders were given an opportunity to provide additional feedback to ensure the report’s accuracy. During coding and analysis, we held frequent meetings to discuss the results and interpretation of the data. Additionally, regular discussions between the authors and other members of the case study team involved in data collection were important to maintain trustworthiness. The study team triangulated their findings by presenting and gathering feedback from other members of the case study team and members of the larger NIH Diversity Program Consortium Coordination and Evaluation Center (CEC) group, who were familiar with each site’s initiatives and practices.

### 4.5 Positionality

It is important to acknowledge that our personal identities and lived experiences inform our viewpoint and analysis of the data. As higher education researchers, our collective interest to ensure equitable access and opportunities for STEMM students informs our perspective. All authors identify as Latinx and/or multiracial and each identifies as first-generation college students. Additionally, one of the authors has a bachelor’s degree in mathematics and understands from first-hand experiences the challenges marginalized communities can face in STEMM. Our identities and experiences as members of these communities inform our interest in promoting STEMM equity. All co-authors of this study were members of the CEC, the larger evaluation body for the BUILD sites. As such, we hold a unique perspective in observing BUILD site student financial support structures. None of the members of the research team had a connection or affiliation with the sites. As outsiders, the research team was able to question processes and practices that may be considered uninteresting by site participants over time (Dwyer and Buckle, 2009) and gain a better understanding of similarities and differences across each site.

### 4.6 Limitations

The exploratory nature of the data collection process meant there were multiple aims of the site visits. One aim was to gain an overall understanding of how BUILD was implemented at each site, with a focus on the student experience rather than specifically the role of financial support in their career trajectory. As such, future research will need to further examine the long-term impacts of financial support on students’ trajectories, such as graduation rates and enrollment in graduate studies. Second, while student focus groups accounted for some group differences according to class status (i.e., first-year student, sophomore, junior, senior), additional research can further examine group differences. Other members of the CEC are conducting quantitative assessments of long-term impact of BUILD as the project sunsets in 2025 and the role of financial support by class standing. Lastly, each site had flexibility in determining the allocation of funds to students, which led to variations in the amount of financial support students received at each site. To address these variations, the research team drew from observations at site visits and from data on each program’s website to understand commonalities in financial support. Each site offered Scholars tuition support (some paid in full while other sites offered partial support to top off packages), a stipend (often through the form of a paid undergraduate research experience), and conference travel funds. While we could document institutional differences in BUILD funding, the research team lacked precise information on the level of financial support each participant in the focus groups received. Additional research can gather this information and examine the influence of varying financial levels on students’ STEMM pathways.

## 5 Findings

Findings from this study reveal that BUILD financial support often helped students take action in pursuing their college and STEMM interests. Specifically, BUILD funding facilitated college entry and participation in biomedical research training experiences as they moved through these pathways. Although students viewed financial support as beneficial to their academic and professional trajectory, they also noted challenges with financial aid processes on campus.

### 5.1 Biomedical program funding influences college entry

Financial support from the BUILD program influenced entry into a biomedical career path for incoming first-year undergraduate students. Specifically, among the four institutions that recruited first-year students into the BUILD program, funding often tipped students’ college choice decision in favor of enrolling at the BUILD institution. The financial support facilitated students’ college enrollment and pursuit of a biomedical degree. Students often pointed to college affordability concerns as a determining factor in their pursuit of a college education. Incoming first-year students often weighed their personal circumstances with an institution’s financial support, and, in many cases, BUILD funding met a necessary financial gap. For example, a student shared the following:

If I didn’t come to [this institution], I probably would have gone to community college first, because all the schools that I applied to and got in, they didn’t give me scholarships compared to [this institution]. Plus, I was the second kid who my parents were putting in college, and my sister didn’t get any scholarships. [My parents] were, like, “If you don’t get any scholarships, then you have to go to community college.” That’s why I’m here.

This example highlights that many BUILD students at the four BUILD sites with programs that began recruiting students in high school were making college choice decisions based on the incentive of financial support that came with acceptance into both the BUILD program and the institution.

Relatedly, BUILD students perceived differences between their financial situation and their peers. Here, a student shared how the financial support received from BUILD benefited their college trajectory by reducing their debt burden and increasing their time to focus on academics:

I think [the financial support] really helps with independence, and it is, of course, very useful because other friends are in a lot of debt at this point, and we graduated high school together. We were on the same path, and we are not anymore on the same path because of all the debt and having to work while going to college. So, their grades aren’t as good. They don’t have the time to really work on that. So that is kind of a plus that we get [from BUILD financial support].

Concerns about debt led some students to choose a college that provided the best financial support. Reductions in costs and debt were viewed as positively contributing to their experience. By selecting a college that provided BUILD funding, students not only alleviated financial concerns, but they perceived differential outcomes in their academic success when compared to their peers. Students viewed the need to work to pay for college costs as consequential to their academic performance and their trajectory. BUILD students perceived that their good grades and ability to remain on their academic and career trajectory were in part due to the financial support they received. Adequate financial support enabled STEMM aspirants in the BUILD programs to pursue their college goals.

Other students perceived that BUILD funding addressed both their affordability concerns and STEMM interests. Students with a strong interest in a STEMM-related career expressed that they weighed both the financial benefits *and* the opportunity to pursue research in the college choice process. For example, a student shared:

A factor that contributed to me coming to [this institution] was also graduating from an early college high school [allowing enrollment at the University]. I wanted to finish my college career in 2 years, my bachelor’s. The biggest factor also was the BUILD scholarship. I always wanted to do research, and BUILD was the perfect opportunity to start my research career.

Financial resources facilitated students’ ability to pursue a STEMM-related career pathway. The added financial resources (e.g., tuition, stipend) for prospective first-year students reduced financial burdens and encouraged them to not only enroll at the institution but also to participate in BUILD. Without this institutional support, students might otherwise have chosen a lower-cost institution despite an interest in the BUILD research training program.

In sum, across the four institutions that recruited incoming first-year students into the BUILD program, the student participants viewed BUILD funding as an initial gateway toward their academic, personal, and professional goals. In several cases, funding from the BUILD program was pivotal to students’ entry into college, undergraduate STEMM programs, and research. BUILD funding consistently reduced or alleviated students’ financial concerns and often informed their decision to enroll in the BUILD institution and participate in the research training program. Incoming first-year students weighed affordability along with their STEMM research interests in their college choice and often pointed to BUILD funds as an important factor in their academic decisions.

### 5.2 Financial resources influence participation in research training

One of the most notable influences of BUILD funding was on students’ entry into and decision to pursue biomedical research activities and experiences, like internships, that are critical for graduate school competitiveness and science identity development (Byars-Winston and Rogers, 2019; Eagan et al., 2024; Estrada et al., 2018). Students described how tuition remission and stipends enabled them to participate in activities related to their STEMM goals. Students’ decisions to participate in the BUILD program, in particular, often resulted from weighing their STEMM-related interests and goals with the full range of academic, professional, and financial support offered by the program. Across all 10 sites, students typically shared that while already coming into college with an interest in STEMM, they were drawn to apply to the BUILD scholarship program primarily because of the program’s wraparound or integrated support services (e.g., cohort activities, advising) and opportunities for research experience. They described BUILD funding as a secondary benefit but not the main reason that initiated their effort to apply to the program. Once in the program, students gained undergraduate research experience and engaged in other career development activities that their BUILD program provided. By offsetting students’ need to work at non-STEMM-related part-time jobs, BUILD students could work in a research setting that aligned with their STEMM interests.

BUILD funding also tipped students’ STEMM training choice by enabling them to explore how societal problems of personal interest could be addressed via biomedical research. For example, a student shared what led to their participation in BUILD:

I wasn’t sure what research is all about, but I did know that the Pacific Islands have an extremely high prevalence of obesity and diabetes rate, as well as cancer and other non-communicable diseases. I want to do something about it because a lot of the people I am close to have been passing away. Every month, I hear somebody who I am close to or a distant relative that passes away on our island, so with [BUILD], since not many people know about the Pacific Islanders or not much research has been done, I decided to go for it and see the advantages of being a Scholar, like they pay for tuition and they covered a lot and give us so much support.

Thus, BUILD program support enabled the student to transform a personal interest in their community’s public health into academic and research goals, which is supported in research about the importance of communal goals (Diekman et al., 2011). Specifically, the student saw how the program supported both tangible financial needs as well as familial and community needs. Participation in the program created an environment in which students could learn about their biomedical research interests and participate in research critical to the health of their own communities.

Similarly, financial support made exploring and pursuing research experiences a viable option for students still assessing their STEMM interests, including deciding whether they enjoy doing research. Students who were unsure of their interest in STEMM research pointed to BUILD funding as a reason for pursuing research training. A student shared:

I heard about BUILD my freshman year, but I didn’t apply for whatever reason until my second year. I guess I just thought about it and then, I kind of went into research thinking maybe I’ll like it, maybe I won’t, and I kind of needed a semester to finish my major here. So why not get paid for it if I get in? That’d be cool, and yeah, I think I’ve enjoyed it so far.

Being paid to do research meant students could satisfy their academic requirements while deciding if STEMM research was a long-term interest. Participating in BUILD led some students to solidify their interests in biomedical research. Although some students viewed BUILD funding as a bonus, the additional financial support remained effective in drawing students into STEMM research. Such insight suggests that addressing the financial needs of students, including those from underrepresented backgrounds, can influence them to move from curiosity to exploring interests and, potentially, making the decision to pursue a STEMM research pathway.

### 5.3 Financial support facilitates pursuit of advanced biomedical training

Financial support also impacted students’ ability to pursue additional research training experiences. In particular, students said that seeking internships, especially those located out of state, often felt out of reach due to associated travel costs. Several BUILD sites allocated funding to cover students’ travel expenses and to mitigate financial concerns. By removing these barriers, students could expand their professional networks and their exposure to biomedical research. As a result of the BUILD program, one student chose a graduate studies program, explaining:

I found this lab…in Washington. BUILD has funds set aside so that you can make your own program. As long as there’s a spot available, there’s funds. You can go somewhere else and work in a research lab over the summer. I contacted Dr. Wallace, and she let me in. I worked with doctors, PhD candidates, and a master student in this one very specific field, and it was absolutely amazing.

The student goes on to say they applied for the doctoral program in Washington due to their positive summer research experience. The student attributed BUILD as a catalyst for their admission to graduate school explaining “being accepted into the doctoral program [would be] impossible without the BUILD Program.” The student perceived they “couldn’t fly to Washington” for the summer internship due to the associated travel costs. Had it not been for the BUILD travel funds, the student believed they would have simply chosen to:

Walk off with my BA and then work at [office name] and do something tangentially related to what I wanted to do, or just give up and become a web developer or something. [The] BUILD program was absolutely instrumental in making [graduate study] happen.

The financial support students received was critical to students’ career goals. In this case, the student could participate in an outof-state summer research experience, which facilitated connections with the faculty and enhanced the student’s interest in the Ph.D. program. Without the funds, students may have been diverted from a STEMM career path despite their interest in pursuing a STEMM research career.

For other students, BUILD support meant they could gain additional career development experiences and build their professional networks through conference attendance. In particular, financial support removed barriers for students seeking to attend biomedical-related conferences. Participation in conferences enables students to disseminate their research, develop their networks, and engage in science socialization, all factors that help bolster science identity (Carlone and Johnson, 2007; Eagan et al., 2024) and research self-efficacy (Bakken et al., 2010), and can strengthen intentions to pursue a science career (Carpi et al., 2017). A student shared the benefits of conference participation:

Definitely, the opportunity to travel for science and to network with other individuals [was a benefit]. And to share your own science; I think it’s huge for development as a researcher to be able to communicate your science to others. I think there’s been a lack of that in the scientific community, and we’re kind of starting to jump over that hurdle now to provide digestible information. So, the financial part of being able to travel with NIH’s support has been huge.

Aid from the BUILD program exposes students to members of biomedical fields and fosters research self-efficacy. Attending conferences resulted in some students connecting with faculty from other institutions and finding summer internship opportunities and additional career preparation. For other students, presenting their research at conferences gave them added confidence in disseminating research. The summer research experiences were often accompanied by financial support provided by the BUILD program or the host site and enabled students to gain additional research training.

### 5.4 Challenges and limitations of grant support

Although students derived many benefits from BUILD financial support, there were notable challenges and limitations to the funding and implementation processes. For many students, the funding alleviated financial concerns and improved college affordability; however, for some students the funding was still not enough to meet cost of living and transportation expenses. For those who were not fully stipended with grants, remaining financial concerns led some students to take on additional employment to make ends meet. Their time and effort spent on working other jobs, in turn, impacted their ability to dedicate time to lab work, courses, and extracurricular activities. A student shared that the need arose to work another job on top of the lab hours required for BUILD, because BUILD funds were insufficient to cover living expenses. In particular, some BUILD institutions are located in areas with a higher cost of living compared to other sites’ geographic locations. A student shared:

While BUILD stipends [are] very helpful, it’s not enough for me to cover my living expenses, like it’s not even close to enough to cover my living expenses so I still have to work about 24–32 hours a week. And so, in addition to my lab work and [in] addition to class work, in addition to my involvement within the psychology department, I don’t get days off for months. I obviously appreciate that I don’t have to pay tuition, but I have to live and the stipend just isn’t enough for that.

Although tuition remission and stipends were meant to reduce or eliminate the need for additional employment, financial barriers continued for some low-income students and undermined the intentions of the BUILD funding. Limitations in funding meant some students took on additional employment to meet their cost-of-living expenses. While some students made the decision to work to address remaining financial gaps, not all students had the opportunity to do so given site-specific program requirements that prohibited them from taking on additional jobs. In those cases, students were frustrated by BUILD program limitations on their ability to work outside of their involvement with BUILD. This finding suggests that while BUILD funding is beneficial, it is not challenge-free and requires examining institutional processes and allocations.

In some instances, institutional processes and requirements regarding financial aid posed hardships for students. Some students were unsure when they would receive their stipends, which affected their ability to pay for expenses. Other students felt ill-informed on financial aid policies and needed to be made aware that accepting stipends from the BUILD program would impact their financial aid package. In particular, some students were unaware that accepting BUILD funding would lead to a reduction in other scholarship awards. Consequently, students who saw a reduction in their financial aid package found they still had a financial gap. Additionally, students were not always aware that their financial aid was taxable income. One student who attended a BUILD site that offered partial tuition remission shared the following:

When I came here and I accepted to be in [BUILD Program], I checked my school bill. I realized they took out all my scholarships. None of them applied. I was just left with the 60% [tuition remission] and it wasn’t even enough. I had to take money out of my own pocket and pay in. It really messed me up. And I had to pay the IRS [Internal Revenue Service] because of [BUILD Program], and I felt like that was very wrong. They didn’t explain that part to me when I came to advising. No one really explained that to me, I got hit with the whole tax [burden].

Although financial support was intended to mitigate these very barriers and to enable students to focus on their coursework, gain research experience, and reduce their financial concerns, many students continued to face income shortfalls and challenges. These challenges point to a need for research training programs such as BUILD to better educate students on the impact of program funding on their overall financial aid awards. Such efforts may require that programs foster stronger partnerships with financial aid offices. Programs must also revisit funding award processes and explore alternative processes for awarding funds if their intent is to supplement rather than replace financial awards. For example, one site hired students under a different classification, thus stretching the grant funds, providing a consistent funding process for BUILD student participants, and maintaining alignment with their own campus and NIH regulations. Without these mechanisms in place, students may continue to experience financial gaps and hardships.

Financial pressures also affected students’ persistence in the BUILD program. More specifically, a few students expressed concerns about losing BUILD funding and perceived they would fare better financially by remaining in the program. For example, one participant shared a default need to stay in the program because the student “could not afford to go anywhere else.” Although this student had contemplated leaving the BUILD program due to its emphasis on earning a Ph.D. and little initial support for students interested in medical degrees, the accompanying financial concerns informed the decision to remain in the program. This suggests that BUILD funds fill an important financial gap among students participating in the program and are important in student persistence in the program. This also demonstrates that BUILD funds contribute to persistence when programs expand support to students interested in exploring other degrees (e.g., medical). Similarly, another student shared concerns about not meeting program requirements and the potential financial repercussions:

That’s what is kind of terrifying here. For example, if you miss two workshops, they kind of dangle your livelihood/your job and your friends over your head, which is a little threatening, because, for example, the program has done a lot for me in the form of I get to talk to people within my community and I have personal development opportunities. I wouldn’t be in my research lab had I not been in the program. Again, it’s kind of terrifying just because you miss two workshops.

Concerns about the financial consequences of leaving the program or not meeting program requirements motivated some students to remain in the BUILD program rather than the BUILD experience itself. Program requirements, such as attendance in this case, are important for ensuring student participation; however, these students’ concerns suggest a need to be more sensitive to the financial concerns of the population that STEMM programs such as BUILD are designed to serve. These requirements may be detrimental to some students and may require additional flexibility or probationary periods for students who do not meet program requirements.

## 6 Discussion and implications

Overall, this study found that financial support from the BUILD program helped convert STEMM interests and goals into actionable steps, sometimes starting as soon as college choice decisions and continuing later into students’ undergraduate trajectories. These findings illustrate the role of financial support within the context of a grant-funded undergraduate biomedical training program on students’ STEMM biomedical career pathways. Employing SCCT, we examined how financial support serves as a contextual influence on biomedical students’ interests, goals, and actions while they were participating in the BUILD program. Overall, contextual influences are not as well-studied as other aspects of the SCCT model (Lent et al., 2000). Efforts to understand the role of contextual influences have examined background contexts such as parental involvement (Byars-Winston and Fouad, 2008) and institutional experiences such as peer support (Lent et al., 2008) and research (Byars-Winston et al., 2016). Thus, this study is unique in that it focuses on the contextual influence of financial support. We explored the meaning students attribute to BUILD funding as it relates to their biomedical training experiences and how it shaped their decisions and trajectories. Studies often use quantitative models to predict outcomes with SCCT as a conceptual framework; however, this study employs a qualitative approach to understand the nuanced relationship between the institutional context and student career interests, goals, and actions.

While it is well known that underrepresented and low-income students weigh college costs in their college decision process (Cox, 2016; Foltz et al., 2014), this study affirmed that BUILD funding was critical for recruiting first-year students into STEMM training programs. Student insights revealed that financial support from the BUILD program was a tipping point factor in their college choice decision. BUILD programs had flexibility in their program structures, and four of the ten BUILD sites recruited high school students and started their BUILD program as early as the summer before the first year of college. The remaining programs recruited students into the program between the sophomore and senior years. While research on college recruitment behavior examines how minoritized students are often excluded from targeted recruitment efforts (Jaquette and Han, 2020), this study found that some BUILD sites leveraged their institutions’ recruitment process to diversify their applicants (for example, outreach to low-income students from underrepresented groups), and succeeded in getting them to choose to attend their institutions to participate in the BUILD program. This finding illuminates the potential for intervention programs to utilize targeted college recruitment efforts to attract incoming talented students from underrepresented groups interested in STEMM disciplines. BUILD was also pivotal in converting students’ STEMM and research interests into a decision to participate in the program. In several instances, tuition remission and a stipend became a determining factor but not the sole factor in students’ decisions to participate in a STEMM training program. Students who chose to participate in BUILD were seeking a holistic experience that addressed their financial, academic, and career needs. This indicates that funding alone might not be enough for students interested in STEMM fields to pursue biomedical research careers (Anderson, 2020). In other words, our study found that students who demonstrated an interest in STEMM research were interested not only in the financial support but also chose to participate in the BUILD program due to the wraparound support provided by the program.

Funding also removed financial barriers related to participation in advanced STEMM research training. Underrepresented and low-income students often face financial barriers to participation in STEMM-related activities (Estrada et al., 2016). The BUILD program sought to remove these barriers and improve engagement in research and professional development activities. Financial support from research training programs can ease financial burdens and improve involvement in STEMM programs (Cobian et al., 2024; Eagan et al., 2024; Stolle-McAllister et al., 2011). In our study, several students indicated that travel support from the BUILD program enabled them to attend conferences and participate in off-site internships. Such funding meant that students interested in pursuing additional research training could participate without the concern of the added costs. Participation in research and professional development activities helped students gain added research experience, build professional networks, and become more competitive for graduate school admission.

While funding removed various cost-related barriers, the amount and process were not without limitations and challenges. Several students indicated that while tuition remission and stipends reduced college costs, many found that the amount they received was insufficient to cover all of their expenses. Tuition remission and stipends effectively reduced college attendance costs; however, the funding did not always address all of the financial stressors and costs students faced. Although some students sought additional employment, program restrictions at some BUILD sites meant that students could not hold another job. This finding suggests that program requirements and financial processes also contributed to ongoing financial concerns. Namely, feeling ill-informed about the financial aid process contributes to financial stressors (Foltz et al., 2014). BUILD students similarly felt that a lack of information on the impact of BUILD funding on financial aid awards and payment processes created additional stress. Funding processes that caused delays in payment at the institution also created additional stress for students. Some students experienced payment delays, which raised serious concerns regarding their ability to make critical payments such as housing costs.

This study provided an opportunity to understand the complexities of financial support for students, including many from groups historically excluded in STEMM. Using SCCT as a lens, we explored students’ meaning-making of their STEMM career goals and actions with a focus on examining how financial support helped or hindered their career-related beliefs and choices. SCCT suggests that certain factors in a person’s life will carry different causal weight in predicting career behavior (Lent et al., 1994). For example, socioeconomic conditions can affect career choices via impact on other elements of SCCT, such as learning opportunities (Lent et al., 1994). We found evidence of financial support being a contextual support, and the process and lack of transparency regarding funding to be a contextual barrier that negatively impacted students’ learning and training experiences. These challenges led to students diverting their time and attention away from biomedical training experiences to attend to financial needs.

### 6.1 Implications for practice and research

Insights from this study provide a more nuanced understanding of the impact of financial support on STEMM students’ academic and career decisions and challenges. In this section, we examine several implications for practice and research to aid institutional leaders in designing or improving financial support processes. This is especially critical for institutions interested in diversifying the STEMM college student population.

STEMM research training programs must include financial and wraparound support (e.g., mentoring, advising) if higher education institutions want to attract and retain students, including those from underrepresented groups. Funding can mitigate financial burdens and influence students’ decisions to participate in a research training program; however, our findings suggest that funding is often a tipping factor in the decision-making process among students with a STEMM interest. Students weighed program offerings with their interests and often chose to participate in BUILD because of the added financial benefit. In addition to offering different forms of institutional and programmatic support, program leaders should ensure clear communication of funding, academic, and career support in their recruitment efforts. Programs interested in recruiting students as they enter college are well advised to communicate these different support mechanisms in their recruitment efforts. Students weighed BUILD funding along with the services and research opportunities offered by the program in their decision to enroll at the institution and/or participate in BUILD. A clear understanding of what institutional support students can expect can contribute to their enrollment decisions and help diversify the STEMM population.

Institutions would do well to ensure all staff and mechanisms are in place to provide adequate student support and timely disbursement of funds. We also acknowledge the budgetary challenges that institutions face as they balance money, training, and staff time devoted to supporting students’ financial needs. Having staff with knowledge about financial aid processes is critical to helping students understand the funding processes and how to best manage their funds. It is especially critical for first-generation college students who rely on campus-based support systems to help them navigate new and unfamiliar processes (Bettencourt et al., 2020). When funding distribution processes were not correctly in place at BUILD sites, these became bureaucratic challenges, which often meant students could not pay expenses, such as housing costs, in a timely manner and they had to make difficult decisions that could have been avoided.

Intervention programs should also provide grant recipients with strategies for navigating the financial systems at their institutions and identifying additional financial support to address any shortcomings. Concerted partnerships between targeted initiatives such as BUILD and financial aid offices can help address gaps in student support. Financial literacy training and support must be paired with funding awards. Students felt better equipped and supported when financial aid policies were explained. Addressing topics such as the payment process, how often students can expect to be paid, and how to plan for future tax deductions will better equip students to manage their funding. Additionally, financial aid directors and STEMM training program coordinators should consider their own policies and practices so that, consistent with funding agencies’ regulation, funding from intervention programs can be considered supplemental rather than a substitute for other forms of institutional aid. Reductions of need-based aid, such as scholarships, from the overall financial aid package often mean students continue to face financial stressors, which undermined the intent of BUILD funding to alleviate financial barriers and concerns.

There are several additional opportunities for future research to fill knowledge gaps. This study attempted to expand our understanding of the contextual influence dimension of SCCT on students’ career decision-making processes. Insights from this study offer a more nuanced understanding of how contextual influences, in this case, financial support, can impact students’ interests and actions. While several of our participants described having STEMM career goals, our study did not indicate if financial support influenced converting STEMM interests into goals. Future studies can further examine the role of financial support in converting interests into goals. For example, future research should adopt a qualitative design that tracks individual students over time, interviewing them at different stages, and comparing their perspectives at the start and end of their participation in targeted initiatives like BUILD. By focusing on individual students over time, researchers can capture the evolving impact of financial factors and provide insights into how such support affects both short-term and long-term STEMM academic and career pathways. Future research should also examine potential disparities for students as a result of program requirements. For some students, program requirements served as both an incentive and a stressor which requires further examination to understand how programs can best offer flexibility to students while achieving program goals. Such insights can point to potential inequities in student participation in research training programs. Additionally, it is essential to examine further the relationship between the dollar amount students receive and their financial concerns. For some students, the amount received appeared to alleviate additional financial concerns, while others continued to experience stressors. Further exploration of the role of context on financial concerns can illuminate if there is an ideal threshold that can fully alleviate student concerns.

Research should also continue examining the role of institutional context in STEMM career pathways. In particular, research should follow students post-graduation and determine the long-term effects of support program funding on students’ STEMM career pathways. How do students’ decisions based on the financial support they receive during their undergraduate studies affect their experiences and decisions after graduation? Future studies can examine the influence of financial support on longer term outcomes, such as students’ decision to pursue graduate studies and the likelihood of persisting in a STEMM career.

## 7 Conclusion

This study produced evidence that financial support shapes critical biomedical training and research career decisions throughout college, from influencing college choice decisions to providing opportunities to strengthen STEMM skills and become socialized into STEMM fields. In practice, BUILD funding chipped away at students’ financial concerns; however, for some students, their need exceeded what was offered by BUILD and these students continued to face financial challenges. Effective STEMM intervention programs must ensure institutional funding processes do not hinder the effects of an intervention for student success. This finding calls for intervention programs to work with the institution to close financial gaps and address processes that may hinder students’ persistence in STEMM.

Ultimately, research must continue to examine the role of funding for students interested in STEMM-related careers, especially those from backgrounds traditionally underrepresented in STEMM. Future studies must consider not only how funding shapes entry into college and research training programs but also how it influences decisions related to course selection, choice of academic major, participation in research experiences, and future graduate studies. A more nuanced understanding of the financial stressors and decision-making process STEMM students face can lead to better policies and practices that comprehensively support underrepresented student populations.

## Data Availability

The datasets presented in this article are not readily available because BUILD site names are publicly available and would affect the confidentiality of participants. Requests to access the datasets should be directed to the Coordination and Evaluation Center, info@diversityprogramconsortium.org.

## Footnotes

1 This study focuses on students in biomedical disciplines with majors in the biological and life sciences, engineering, physical sciences, health professions, social sciences, and computer science.

2 We use the term “Latine” as a gender-neutral term to refer to individuals of Latin American heritage.

3 Four students reported double majors in different categories and are counted twice; therefore, the total percentage may not add up to 100%.
